# Supplementary material for: Chromothripsis during telomere crisis is independent of NHEJ, and consistent with a replicative origin
Source: Genome Res. 2019 May;29(5):737–49. doi: 10.1101/gr.240705.118 (PMC6499312; doi:10.1101/gr.240705.118)
Supplement: Supplemental Material [file supp_gr.240705.118_Supplemental_file_1.zip › contigs/annotated_contigs/DB48/contig.2.DB48_length_532_mean_cov_5.69736842105.docx]

**DB48_length_532_mean_cov_5.69736842105**

```
TACAT|GTGTTGAAATGGCAAAGTAGCCTTTAAATAACAAAGCATAAAGAAAATAGTGACTTGAGTTTCTTGAAGAACCATACACTTTC
      >chr10:27244583-27244858 + E=7e-154
TAAGGGATATTAGTCCTGTGTATGCAACAAGTGAGAACATTAAGAAGGCTGTTTTATTTTATCAGCTGTGGTTCCTGTTGATCCTGTGG

AAGAAAATTATGTTCATAAAGTGGAAACTGTATTTTTTTCAATCTCCTTCCCAACTCCTTTCAAATCAAGAGTACGTCTTGTAGCAGTT

TCAAAGGTTAGG|CA|GAAAATTTCTGTGGGTTATTGCTAGAGAACTCATTCAGTGCACTTTTGGCAAGAGATTTTGGCAAAATGAAGC
             >chr10:27245948-27246202 + E=3e-141
AGTACAACATGTCAGTATAAAAGGACAAATAAGCCAGGTGCGGTGGCTCACGCCTATAATCCCAGCATTTTGTGAGGCAGAGGCAGGCA

GATAGCTTGAGACTAGGAGTTCAAGACCAGCCTGGGCAACATGGCTGTACAAAAATTAGCCAGGCATAGTGATGTGTGCCTGTGGTCCC

A
```
